# Autophagy-Virus Interplay: From Cell Biology to Human Disease

**DOI:** 10.3389/fcell.2018.00155

**Published:** 2018-11-19

**Authors:** Liyana Ahmad, Serge Mostowy, Vanessa Sancho-Shimizu

**Affiliations:** ^1^Department of Virology, Division of Medicine, Imperial College London, London, United Kingdom; ^2^MRC Centre of Molecular Bacteriology and Infection (CMBI), Imperial College London, London, United Kingdom; ^3^Department of Immunology and Infection, London School of Hygiene and Tropical Medicine, London, United Kingdom; ^4^Department of Paediatrics, Division of Medicine, Imperial College London, London, United Kingdom

**Keywords:** autophagy, inflammation, HIV-1, HSV-1, IAV, viral immunity

## Abstract

Autophagy is a highly conserved intracellular degradation process that targets protein aggregates and damaged organelles. Autophagy is also implicated in numerous viral infections, including human immunodeficiency virus-1 (HIV-1), influenza A (IAV) and herpes simplex virus-1 (HSV-1). Depending on the virus, autophagy can restrict or promote viral replication, and play key roles in modulating inflammation and cell survival. In this review, we consider examples of autophagy-virus interplay, highlighting the protective role of autophagy in human infections. We summarize recent discoveries and emerging themes illuminating autophagy’s role in immunity and inflammation upon viral infection. Finally, we discuss future prospects and therapeutic implications, and potential caveats associated with using autophagy to control viral infections in humans.

## Introduction

**FIGURE 1:**
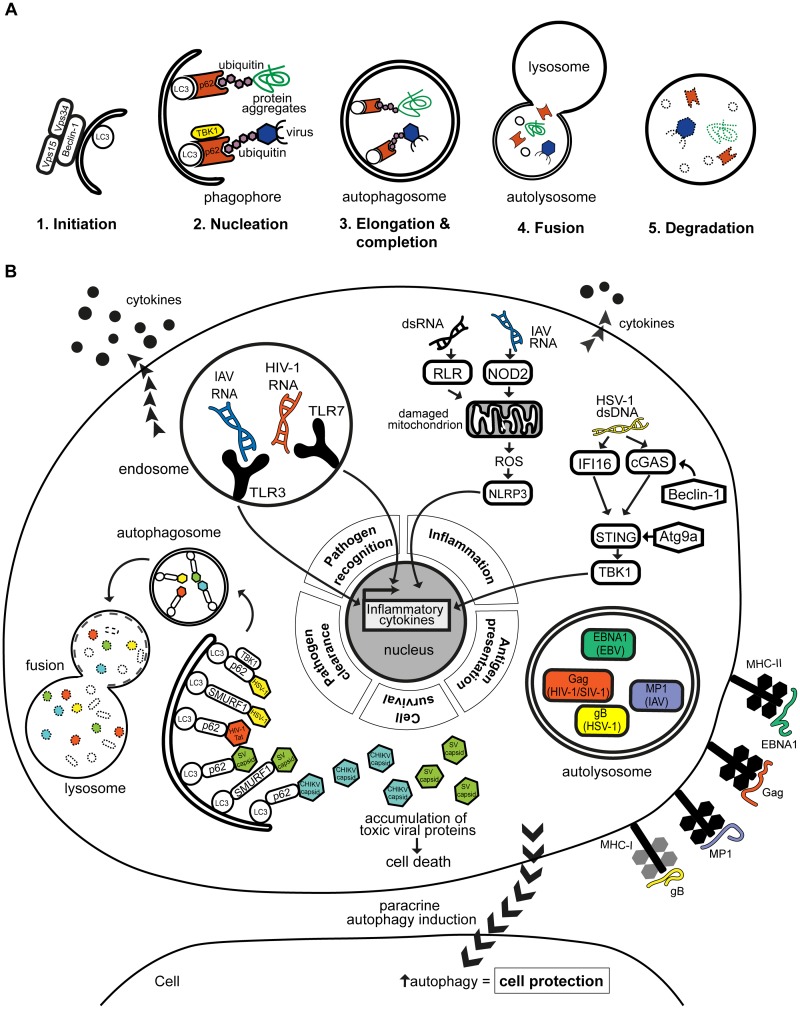
**(A)** Autophagy is a regulated multi-step process that leads to cargo degradation. Autophagy can eliminate cargo such as virus and viral-derived antigens. It can be organized into 5 distinct steps beginning with (1) the initiation of phagophore formation which (2) nucleates around the intended cargo. The cargo can be selectively recruited by autophagy receptors such as p62, which can be regulated by TBK1. (3) The phagophore elongates and completes to form a structure termed autophagosome which then (4) fuses with nearby lysosomes carrying hydrolytic enzymes. This eventually leads to (5) the acidification and hence degradation of the contained cargo. **(B)** Autophagy plays an antiviral role in various human infections by modulating different aspects of the immune response. Autophagy facilitates viral clearance by recruiting selective autophagy receptors p62 and SMURF1 to target viral components to autophagosomes for lysosomal degradation. Reported targets include HSV-1, HIV-1 Tat protein, and the capsids of CHIKV and SV. Disruption of the targeting of viral proteins, such as CHIKV and SV capsids, may lead to their toxic accumulation and cause cell death. Autophagy also promotes pathogen recognition by aiding delivery of viral PAMPs, e.g., HIV-1 and IAV RNA genomes to cognate TLRs in endosomes, which results in enhanced production of antiviral cytokines. On the other hand, autophagy can prevent excessive inflammation by negatively regulating signaling pathways through Atg9a or Beclin-1, or by clearing mitochondria that are producing inflammatory-inducing signals such as reactive oxygen species (ROS). Autophagy supports amplification of inflammatory responses by regulating adaptive immune responses, through the processing and presentation of viral antigens, such as EBV EBNA1, IAV MP1, HIV-1/SIV gag and HSV-1 glycoprotein B, on MHC class I or II to T cells. Autophagy can also be induced in distant cells, i.e., in paracrine manner, which may confer protection to these cells as seen with multiple viral infections including CVB, HCV, and HSV-1.

Autophagy captures cytoplasmic contents, such as excess or defective proteins and organelles, for degradation by the lysosome. It is initiated in response to various stimuli, including nutritional state of the cell and environmental stresses such as starvation and hypoxia. As such, autophagy is an important process of regulating cellular homeostasis and survival. It is a well-studied process that is orchestrated by over 35 autophagy-related (ATG) proteins and can be organized into multiple steps: phagophore initiation, membrane elongation, autophagosome formation and autophagosome fusion with hydrolytic lysosomes (Figure 1A; Mizushima et al., 2011). Autophagy can be selective in terms of cargo capture via the recruitment of selective autophagy receptors. Autophagy receptors can interact with ubiquitin tags that decorate the cargo [via its ubiquitin-binding domain (UBD)], and with LC3 proteins of nascent autophagosomes [via its LC3-interacting region (LIR) motif] (Stolz et al., 2014). Some autophagy receptors, particularly p62/SQSTM1 and optineurin, are regulated by Tank-binding kinase 1 (TBK1)-mediated phosphorylation, and are key players in the autophagic degradation of invasive pathogens (Wild et al., 2011; Pilli et al., 2012; Sparrer et al., 2017).

Autophagy plays a key role in cellular immunity to human infections (Randow et al., 2013; Paul and Münz, 2016). In the case of human viral infections, autophagy can be either proviral or antiviral (Levine et al., 2011; Jackson, 2015). Some viruses highjack the autophagy machinery for their intracellular survival, while others express specific proteins to evade autophagy and propagate in host cells. Antiviral autophagy can (1) selectively target pathogens for degradation, (2) promote pathogen recognition and inflammatory cytokine responses, (3) regulate inflammation, (4) control cell survival, (5) promote antigen presentation and/or (6) be regulated in a paracrine-mediated fashion, a departure from the classic cell autonomous route (Figure 1B; Levine, 2005; Levine et al., 2011; Jackson, 2015; Paul and Münz, 2016). By focusing on these themes of antiviral autophagy, this review will highlight the protective nature of autophagy. We will draw from examples of human viral infections representing significant disease burden, whose interplay with autophagy has been supported by experimental and/or clinical evidence.

## Pathogen or Viral Antigen Clearance by Autophagy

Selective autophagy has been reported to control several human viral infections *in vitro*, leading to the clearance of pathogens or viral antigens and host cell survival. One of the first viruses shown to engage the autophagy machinery was Sindbis virus (SV), a positive-stranded RNA alphavirus that typically causes mild disease in humans. Autophagy receptors p62 and SMURF1 were found to target SV capsid, in a ubiquitin-independent manner, to autophagosomes in human HeLa cells and mouse embryonic fibroblasts (MEFs) (Orvedahl et al., 2010, 2011). Depletion of p62 in HeLa cells or *Atg5* in mouse neurons *in vivo* led to an accumulation of toxic SV capsid and higher virus-induced mortality, without altering SV replication (Orvedahl et al., 2010). Whether SV can express proteins to counteract autophagy is not yet known.

Similar to SV, p62 is involved in targeting toxic Chikungunya virus (CHIKV) capsid to autophagosomes in HeLa cells, but in a ubiquitin-dependent manner (Judith et al., 2013). CHIKV, also a positive-stranded RNA alphavirus, is a mosquito-borne virus causing severe pathologies in humans that range from febrile arthralgia, rash to encephalopathy (Couderc and Lecuit, 2015). siRNA-mediated depletion of p62 led to an increase in both cell mortality and CHIKV replication, while p62 overexpression promoted cell viability in CHIKV-infected cells (Judith et al., 2013). In contrast, another autophagy receptor NDP52 was shown to have a proviral role although overall autophagy played a cytoprotective role in CHIKV infection (Judith et al., 2013).

Human immunodeficiency virus-1 (HIV-1), a lentivirus that is transmitted sexually through infected body fluids, is targeted by autophagy in human CD4^+^ T cells *in vitro* (Sagnier et al., 2015). HIV-1 primarily infects CD4^+^ T cells, consequently compromising the individual’s immune defense and leading to acquired immunodeficiency syndrome (AIDS) (Maartens et al., 2014). HIV-1 integrates its DNA into the host’s genome, and recruits its transactivator protein Tat to activate viral transcription. Tat protein is therefore essential for HIV-1 replication. However, Tat protein is targeted by p62 in HEK293T cells, which directs it to autophagosomes in a ubiquitin-independent manner (Sagnier et al., 2015). Consistent with this, depleting p62 in HEK293T cells resulted in accumulation of Tat protein, and enhancing autophagy in a chronically HIV-1-infected T cell line led to reduction of Tat protein levels (Sagnier et al., 2015). Furthermore, peripheral blood mononuclear cells (PBMCs) from HIV-1-infected non-progressor individuals showed higher number of HIV-1 particle-containing autophagic vesicles compared with HIV-1-infected normal progressors, suggesting a role for autophagy in limiting the pathogenesis of HIV-1 *in vivo* (Nardacci et al., 2014). However, HIV-1 can modulate autophagy and expresses multiple autophagy inhibitors, such as Vif, Nef, and Env, which operate in a cell type-specific manner (Liu et al., 2017). Hence, autophagy can be viewed to control HIV-1 replication by targeting viral components to degradation in specific cell types.

Herpes simplex virus-1 (HSV-1) is a ubiquitous, neurotropic α-herpesvirus with a global seroprevalence of 67% (Looker et al., 2015). It typically manifests as benign, self-limiting mucocutaneous ulcers, but in rare cases may cause life-threatening herpes simplex encephalitis (HSE) (Whitley and Roizman, 2001). Reports have identified HSV-1-encoded ICP34.5 and Us11 as autophagy inhibitors, which exert their effects by targeting Beclin-1 and protein kinase R (PKR), respectively (Orvedahl et al., 2007; Lussignol et al., 2013). Studies in primary MEFs and mice described increased autophagy following infection with ICP34.5-deficient HSV-1, as suggested by an increased number of autophagosomes and virions in neuronal autophagosomes, respectively (Tallóczy et al., 2006; Alexander et al., 2007). During HSV-1 infection, autophagy appears to be operating in a cell type-specific manner (Yordy et al., 2012). For example, in contrast to mitotic cells such as MEFs and mouse keratinocytes, post-mitotic mouse primary neuronal cells predominantly use autophagy over interferon (IFN) as a viral control mechanism (Yordy et al., 2012). In MEFs, HSV-1 was shown to be selectively targeted by p62 and SMURF1. HSV-1-infected *smurf1−/−* MEFs failed to target HSV-1 virions to autolysosomes, resulting in more HSV-1 virions in autophagosomes (Orvedahl et al., 2011). In mouse primary trigeminal neurons *in vitro*, HSV-1 infection triggered the formation of p62-mediated autophagosomes (Katzenell and Leib, 2016). Finally, infection of HEK293T cells with ICP34.5-deficient HSV-1 led to the recruitment of TBK1- and p62-mediated autophagy, and viral restriction (Sparrer et al., 2017). However, the exact viral target of p62 and SMURF1, and the role of ubiquitin in mediating receptor-virus interactions, is not yet known. Reports using human HFFF2 fibroblasts and mouse dendritic cells (DCs) have shown that autophagy is triggered by HSV-1 double-stranded DNA (dsDNA), and is independent of viral replication but dependent on STING (McFarlane et al., 2011; Rasmussen et al., 2011).

## Autophagy and Cellular Immunity: Pathogen Recognition and Cytokine Responses

Cellular immunity requires the detection of viral pathogen-associated molecular patterns (PAMPs) by their cognate receptors to produce antiviral cytokines, such as type-I IFNs (Iwasaki, 2012). Autophagy can deliver viral PAMPs to their receptors, and help amplify the production of inflammatory cytokines. The capacity of autophagy to facilitate viral recognition and modulate downstream cytokine production has been demonstrated in the case of HIV-1 infections. During HIV-1 infection in human primary plasmacytoid DCs, autophagy plays a key role in presenting the HIV-1 RNA genome to its cognate immune receptor Toll-like receptor-7 (TLR7) in endosomes, leading to the induction of IFNα (Lee et al., 2007; Zhou et al., 2012). Silencing the expression of ATG7 in plasmacytoid DCs *in vitro* leads to a significant decrease in IFNα production following HIV-1 infection, highlighting a crucial role for autophagy in mediating TLR7-IFN signaling (Zhou et al., 2012).

Autophagy also plays an important role in mediating cytokine production during infection by influenza A virus (IAV). IAV, an RNA virus, is a pandemic threat and global health concern. It targets epithelial cells of the respiratory tract, and in severe cases may cause pneumonia or pulmonary damage (Paules and Subbarao, 2017). In the case of infection with highly virulent IAV strains H1N1 and H5N1, high morbidity has been attributed to excessive host-induced inflammatory cytokine production (Peiris et al., 2010). IAV infection was shown to induce autophagy in primary human blood macrophages, which regulates the production of CXCL10 and IFNα. When these cells were depleted of ATG5 or treated with the autophagy inhibitor 3-methyladenine (3-MA), they produced lower CXCL10 and/or IFNα levels (Law et al., 2010). The precise mechanism by which autophagy facilitates this aspect of IAV infection in human blood macrophages is unknown, but is thought to involve recognition of viral RNA by endosomal TLR3 (Law et al., 2010). In contrast, IAV-induced autophagy in MEFs can prevent IFNβ production, and enhance viral replication (Perot et al., 2018). The induction of autophagy during IAV infection is complex: it is initially cytoprotective but is later counteracted by the IAV matrix protein 2 (M2) which targets Beclin-1 to block lysosomal fusion with autophagosomes (Gannagé et al., 2009). Taken together, these data suggest an immunopathological role of autophagy in controlling cytokine production and IAV infection, in potentially a tissue-specific manner.

From the examples of HIV-1 and IAV, autophagy plays a fundamental role in modulating the primary antiviral response, by promoting viral recognition through TLR-dependent signaling and inflammatory cytokine production.

## Anti-Inflammatory Actions of Autophagy

In addition to promoting inflammation, autophagy is crucial for preventing prolonged and excessive inflammation detrimental to the host. Components of the autophagy machinery, such as Beclin-1 and Atg9a, interact with the cytoplasmic type-I-IFN-inducing STING-TBK1 pathway. A study found that Beclin-1 interacts with dsDNA sensor cGAS to dampen IFNβ production in HEK293T cells stimulated with dsDNA or infected with HSV-1 (Liang et al., 2014). This interaction also leads to clearance of dsDNA through autophagy, limiting the otherwise persistent IFNβ-mediated inflammation (Liang et al., 2014). The depletion of Beclin-1 was shown to increase cGAS-mediated IFNβ production while reducing HSV-1 replication in RAW264.7 mouse macrophages (Liang et al., 2014). In MEFs stimulated with dsDNA, Atg9a had a similar role and negatively regulated the STING-TBK1-IFN pathway by binding to STING and preventing its assembly with TBK1 in LC3-positive structures (Saitoh et al., 2009). *Atg9a*-knockout mice revealed an increase in IRF3 phosphorylation and IFNβ production following dsDNA stimulation (Saitoh et al., 2009). However, whether HSV-1 infection (or infection of other dsDNA viruses) is subject to Atg9a-mediated regulation remains unknown.

Autophagy may also negatively regulate inflammation indirectly by clearing host DAMPs, such as reactive oxygen species (ROS) released by mitochondria. This has been observed in mice BMDCs where IAV genomic RNA is detected by the NOD2-RIPK2 pathway, which activates ULK1 to induce RIPK2-mediated autophagic clearance of damaged mitochondria (Lupfer et al., 2013). In RIPK2-deficient mice BMDCs infected with IAV, mitochondria accumulated in cells resulting in elevated production of superoxide. This led to the hyperactivation of the NLRP3 inflammasome and an increased secretion of the inflammatory cytokine interleukin (IL)-18 (Lupfer et al., 2013). A similar observation was made in *Atg5*-deficient MEFs and mouse primary macrophages stimulated with dsRNA analog poly(I:C), which led to excessive RIG-I-like-receptor signaling (Tal et al., 2009). Furthermore, ectopic P-granules autophagy protein 5 homolog (EPG5; a protein that regulates autolysosomal formation) has been shown to control pulmonary inflammation. Lung macrophages from *Epg5−/−* mice showed excessive production of inflammatory IL-1β and IL-13 cytokines, resulting in resistance to IAV infection (Lu et al., 2016).

These reports highlight the important role of autophagy in attenuating inflammation. The autophagy machinery can limit inflammation by regulating cytosolic NLR- and STING-mediated signaling pathways through disposal of their ligands, inactivation of their cognate receptors or interaction with their downstream effector molecules.

## Autophagy and Cell Survival

As shown from investigations of multiple human viral infections, autophagy plays a role in promoting cell survival and limiting pathogenesis. This has been demonstrated by the ability of mouse L cell mutant gro29 cells which have high basal autophagy to restrict HSV-1 replication (Le Sage and Banfield, 2012). In contrast, Atg16L^HM^ mice, which have reduced basal autophagy, showed high mortality following infection with CHIKV *in vivo* (Joubert et al., 2012). One mechanism by which autophagy may promote cell survival during viral infection is by degrading and preventing accumulation of toxic viral proteins, such as viral capsids, in the infected cells. This has been demonstrated in the case of CHIKV and SV infected cells, as discussed above (Orvedahl et al., 2010; Judith et al., 2013). Furthermore, reports have documented the cytoprotective effect of autophagy-enhancing drugs, such as vitamin D, MG132 and rapamycin, in viral infections. For example, primary human macrophages have shown benefit from vitamin D treatment, which limits HIV-1 replication *in vitro* (Campbell and Spector, 2012). Treating HSV-1-infected human corneal epithelial (HCE) cells with MG132 can reduce viral titres (Yakoub and Shukla, 2015). Moreover, pre-conditioning human fibroblasts *in vitro* with rapamycin has been shown to promote cell survival following HSV-1 infection (Ahmad et al., 2018). This cytoprotective role for autophagy that occurs early in HSV-1 infection appears to be a TBK1-dependent process (Ahmad et al., 2018). In agreement with this, TBK1 deficiencies render human fibroblasts susceptible to HSV-1 infection and leads to increased cell mortality (Herman et al., 2012). These data support literature showing that TBK1 is a key player in protective autophagy against bacterial infections (Weidberg and Elazar, 2011), and extend its protective role to viral infection.

## Autophagy and Adaptive Immunity: Antigen Presentation

Through its degradative function, autophagy is particularly useful for generating endogenous peptide antigens for major histocompatibility complex (MHC)-II presentation (Dengjel et al., 2005; Paul and Münz, 2016). In viral infections, autophagy generates viral antigens loaded onto MHC-I and MHC-II for presentation to T cells (Münz, 2017). Epstein-Barr virus (EBV) is an oncogenic γ-herpesvirus causing a spectrum of human diseases ranging from mononucleosis to lymphomas and carcinomas (Taylor et al., 2015). Historically, EBV nuclear antigen 1 (EBNA1) was one of the first viral antigens shown to be processed by autophagy and loaded on MHC-II molecules of EBV-transformed B cell lines (Paludan et al., 2005). Inhibition of autophagy leads to accumulation of EBNA1 in autophagosomes of EBV-transformed lymphoblastoid cell lines, and a decrease in EBNA1-specific CD4^+^ T cell recognition via MHC-II (Paludan et al., 2005).

As a result of reduced MHC-II antigen presentation, mice with *Atg5*-deficient DCs intradermally injected with HSV-1 showed significantly lower IFNγ production by CD4^+^ T cells (Lee et al., 2010). In addition, autophagy can deliver viral antigens for MHC-I cross-presentation. Using a mouse BMA3.1A7 macrophage cell line for CD8^+^ cell stimulation, HSV-1 glycoprotein B (gB) was presented on MHC-I in an autophagy-dependent manner (English et al., 2009; Radtke et al., 2013).

Autophagy is also vital for efficient stimulation of antiviral CD4^+^ T cells in HIV-1/Simian immunodeficiency virus (SIV) and IAV infections. Knocking down LC3 protein or inhibiting autophagy using 3-MA in human DCs led to reduced antigen processing and MHC-II presentation, and a decrease in HIV-1-specific CD4^+^ T cell response (Blanchet et al., 2010). On the other hand, enhancing autophagy in human DCs with rapamycin resulted in a more pronounced HIV-1-specific CD4^+^ T cell response (Blanchet et al., 2010). Fusing SIV gag protein to LC3 in mice BMDCs was also shown to improve antigen-specific CD4^+^ T cell responses *in vitro* (Jin et al., 2014). Similar results were obtained *in vivo* where immunizing mice with SIV gag-LC3 resulted in a stronger humoral immune response, with CD4^+^ T cells producing higher levels of IFNγ, TNFα and IL-2 (Jin et al., 2014). Conjugating IAV matrix protein 1 (M1) to LC3 in HaCat human epithelial cells, B cells and DCs led to enhanced antigen-specific human CD4^+^ T cell responses *in vitro*, as measured by IFNγ (Schmid et al., 2007).

Taken together, autophagy can perpetuate the initial response to viral infection by priming and mediating T cell responses of the adaptive immune system to ensure effective viral clearance.

## Beyond Cell Autonomous Immunity: Paracrine Regulation of Autophagy

Since its discovery, the primary focus of autophagy research has been to investigate its role on a cell autonomous level. Interestingly, two recent reports have demonstrated that autophagy can also be triggered at a cell population level (i.e., in a paracrine manner) to affect distant cells. A first report showed that autophagy could be triggered in distant and distinct cell types that can protect them from a variety of viral infections (Delorme-Axford et al., 2013). In this case, primary human placental trophoblasts can protect other cells from coxsackievirus B3 (CVB), hepatitis C virus (HCV), vesicular stomatitis virus (VSV) and vaccinia virus (VACV), by secreting signals that induce autophagy to resist infections. This concept is particularly relevant in the womb, allowing maternal trophoblasts to confer resistance to viral infections to the growing fetus.

A second report described the paracrine regulation of autophagy early in HSV-1 infection (Ahmad et al., 2018). In this case, HSV-1 infection of human fibroblasts was shown to induce autophagy in cells neighboring an infection site. Despite having functional basal autophagy, HSE patient-derived fibroblasts deficient in TBK1 specifically failed to mount paracrine-mediated autophagy during HSV-1 infection. The study further showed that autophagy induction early during infection may protect cells from death. The autophagic role of TBK1 has previously been associated with inflammation control in neurodegenerative amyotrophic lateral sclerosis (ALS) (Freischmidt et al., 2015). These observations highlight a potential involvement of TBK1 in controlling neuroinflammation through autophagy in HSE.

## Conclusion

Many open questions remain concerning the precise role of autophagy in human viral infections. Studies looking at human responses *in vivo* are rare, due to difficulty of conducting these studies. However, a wealth of studies using animal *in vitro/ex vivo/in vivo* and human *in vitro/ex vivo* models have given remarkable insights into the role of autophagy in disease manifestation.

In this review, we discuss human viruses modulated by autophagy that represent a significant clinical burden. We highlight how autophagy is protective and may be used to enhance current treatment options (Table 1; Whitley and Roizman, 2001; Moscona, 2005; Maartens et al., 2014; Couderc and Lecuit, 2015). Several reports have shown the protective role of p62-mediated selective autophagy in various human pathogens (e.g., CHIKV, HIV-1, and HSV-1), making p62 an attractive therapeutic target. Enhancement of autophagy through p62 may provide an important therapeutic avenue for treatment of human viral diseases (Table 1). However, p62 also participates in other biological processes, such as cell proliferation and ubiquitin-proteasomal degradation (Liu et al., 2016b), and research focusing on employing p62 for therapeutic benefit should be aware of potential pleiotropic effects. The ability of p62 to interact with viruses independent of its conventional ubiquitin-binding domain also warrants further investigation. As shown in the case of HSV-1 and HIV-1 infections, augmenting autophagy using stimulants (such as rapamycin and vitamin D) can be beneficial to restrict viral replication and/or promote cell survival. Enhancing autophagy in vaccine therapies has also been beneficial, taking advantage of the role of autophagy in antigen priming. Promising results were observed in the case of IAV and HIV-1/SIV-1 infections, whereby increasing the autophagic targeting of viral protein gave rise to a heightened adaptive immune response (Schmid et al., 2007; Jin et al., 2014). Moreover, autophagy is important for both MHC-I and/or -II antigen presentation in HSV-1 and HIV-1 infections, as well as for regulating inflammation by facilitating antiviral inflammatory cytokine production. Autophagy’s role in fine-tuning inflammation is also important during IAV infection, where it promotes inflammatory cytokine production and prevents excessive inflammatory responses. In addition to the effects on acute disease outcome, modulating autophagy may have a promising role in the prevention or treatment of various viral post-infectious inflammation/autoimmune disorders for which there are limited treatment options (Table 1; Kovalevich and Langford, 2012; Armangue et al., 2014; Couderc and Lecuit, 2015; Liu et al., 2016a). Harnessing autophagy’s inflammation-reducing capacity can potentially prevent the development of these states, or help to resolve the inflammatory symptoms.

**Table 1:** Opportunities for autophagy-modifying therapeutic intervention in human viral diseases.

Virus	Available treatment for acute disease	Vaccine	Post-infectious inflammation/autoimmunity
CHIKV	None	No	Rheumatic inflammation
HIV-1	Antiretroviral therapy (ART)	No	HIV-associated neuroinflammation
HSV-1	Nucleoside analog (e.g., acyclovir)	No	Anti-N-methyl-D-aspartate receptor (NMDAR) encephalitis
IAV	Neuraminidase inhibitors (e.g., oseltamivir)	Yes	Acute infection-induced cytokine storm

Current research on autophagy is mostly focused on its role in cell autonomous immunity (Randow et al., 2013). However, recent studies have revealed a novel type of autophagy triggered in a paracrine manner in response to viral infections. Elucidating the role of paracrine-regulated autophagy may prove to be highly relevant in disease pathogenesis *in vivo*, and may be useful as a method of clinical intervention. It is tempting to speculate that similar mechanisms could also be extended to viral pathogenesis that disseminates to sensitive tissues, such as the central nervous system (CNS).

In conclusion, we have discussed here the protective nature of autophagy in light of important human viral infections, and highlighted potential therapeutic strategies that can be pursued through autophagy modulation. Most viral infections result in complex host-pathogen interplay, and therefore routes of intervention require careful consideration in terms of application. For example, certain studies of autophagy in HSV-1 infections have shown viral restriction whilst others have only demonstrated cytoprotective effects despite the presence of viral autophagy inhibitors, which may be partly due to cell type specificity. These studies reveal the intimate interactions of the virus and the host cell which will require further dissection if we wish to target the appropriate molecular pathways for antiviral therapies.

## Author Contributions

LA, SM, and VS-S conceptualized and wrote this review. Figure was prepared by LA. All authors approved the final version of this review and agreed to be accountable for the content of the work.

## Conflict of Interest Statement

The authors declare that the research was conducted in the absence of any commercial or financial relationships that could be construed as a potential conflict of interest.

## References

[B1] Ahmad L. Mashbat B. Leung C. Brookes C. Hamad S. Krokowski S. (2018). Human TBK1 is required for early autophagy induction upon HSV1 infection. *J. Allergy Clin. Immunol.* 10.1016/j.jaci.2018.09.013 [Epub ahead of print]. 30296527

[B2] Alexander D. E. Ward S. L. Mizushima N. Levine B. Leib D. A. (2007). Analysis of the role of autophagy in replication of herpes simplex virus in cell culture. *J. Virol.* 81 12128–12134. 10.1128/JVI.01356-07 17855538 PMC2169004

[B3] Armangue T. Leypoldt F. Málaga I. Raspall-Chaure M. Marti I. Nichter C. (2014). Herpes simplex virus encephalitis is a trigger of brain autoimmunity. *Ann. Neurol.* 75 317–323. 10.1002/ana.24083 24318406 PMC3961499

[B4] Blanchet F. P. Moris A. Nikolic D. S. Lehmann M. Cardinaud S. Stalder R. (2010). Human immunodeficiency virus-1 inhibition of immunoamphisomes in dendritic cells impairs early innate and adaptive immune responses. *Immunity* 32 654–669. 10.1016/j.immuni.2010.04.011 20451412 PMC2929482

[B5] Campbell G. R. Spector S. A. (2012). Vitamin D inhibits human immunodeficiency virus type 1 and *Mycobacterium tuberculosis* infection in macrophages through the induction of autophagy. *PLoS Pathog.* 8:e1002689. 10.1371/journal.ppat.1002689 22589721 PMC3349755

[B6] Couderc T. Lecuit M. (2015). Chikungunya virus pathogenesis: From bedside to bench. *Antiviral Res.* 121 120–131. 10.1016/j.antiviral.2015.07.002 26159730

[B7] Delorme-Axford E. Donker R. B. Mouillet J.-F. Chu T. Bayer A. Ouyang Y. (2013). Human placental trophoblasts confer viral resistance to recipient cells. *Proc. Natl. Acad. Sci. U.S.A.* 110 12048–12053. 10.1073/pnas.1304718110 23818581 PMC3718097

[B8] Dengjel J. Schoor O. Fischer R. Reich M. Kraus M. Müller M. (2005). Autophagy promotes MHC class II presentation of peptides from intracellular source proteins. *Proc. Natl. Acad. Sci. U.S.A.* 102 7922–7927. 10.1073/pnas.0501190102 15894616 PMC1142372

[B9] English L. Chemali M. Duron J. Rondeau C. Laplante A. Gingras D. (2009). Autophagy enhances the presentation of endogenous viral antigens on MHC class I molecules during HSV-1 infection. *Nat. Immunol.* 10 480–487. 10.1038/ni.1720 19305394 PMC3885169

[B10] Freischmidt A. Wieland T. Richter B. Ruf W. Schaeffer V. Müller K. (2015). Haploinsufficiency of TBK1 causes familial ALS and fronto-temporal dementia. *Nat. Neurosci.* 18 631–636. 10.1038/nn.4000 25803835

[B11] Gannagé M. Dormann D. Albrecht R. Dengjel J. Torossi T. Rämer P. C. (2009). Matrix protein 2 of influenza A virus blocks autophagosome fusion with lysosomes. *Cell Host Microbe* 6 367–380. 10.1016/j.chom.2009.09.005 19837376 PMC2774833

[B12] Herman M. Ciancanelli M. Ou Y.-H. Lorenzo L. Klaudel-Dreszler M. Pauwels E. (2012). Heterozygous TBK1 mutations impair TLR3 immunity and underlie herpes simplex encephalitis of childhood. *J. Exp. Med.* 209 1567–1582. 10.1084/jem.20111316 22851595 PMC3428952

[B13] Iwasaki A. (2012). A virological view of innate immune recognition. *Annu. Rev. Microbiol.* 66 177–196. 10.1146/annurev-micro-092611-150203 22994491 PMC3549330

[B14] Jackson W. T. (2015). Viruses and the autophagy pathway. *Virology* 47 450–456. 10.1016/J.VIROL.2015.03.042 25858140 PMC5917100

[B15] Jin Y. Sun C. Feng L. Li P. Xiao L. Ren Y. (2014). Regulation of SIV antigen-specific CD4+ T cellular immunity via autophagosome-mediated MHC II molecule-targeting antigen presentation in mice. *PLoS One* 9:e93143. 10.1371/journal.pone.0093143 24671203 PMC3966893

[B16] Joubert P.-E. Werneke S. W. de la Calle C. Guivel-Benhassine F. Giodini A. Peduto L. (2012). Chikungunya virus–induced autophagy delays caspase-dependent cell death. *J. Exp. Med.* 209 1029–1047. 10.1084/jem.20110996 22508836 PMC3348111

[B17] Judith D. Mostowy S. Bourai M. Gangneux N. Lelek M. Lucas-Hourani M. (2013). Species-specific impact of the autophagy machinery on Chikungunya virus infection. *EMBO Rep.* 14 534–544. 10.1038/embor.2013.51 23619093 PMC3674439

[B18] Katzenell S. Leib D. A. (2016). Herpes simplex virus and interferon signaling induce novel autophagic clusters in sensory neurons. *J. Virol.* 90 4706–4719. 10.1128/JVI.02908-15 26912623 PMC4836354

[B19] Kovalevich J. Langford D. (2012). Neuronal toxicity in HIV CNS disease. *Future Virol.* 7 687–698. 10.2217/fvl.12.57 23616788 PMC3632417

[B20] Law A. H.-Y. Lee D. C.-W. Yuen K.-Y. Peiris M. Lau A. S.-Y. (2010). Cellular response to influenza virus infection: a potential role for autophagy in CXCL10 and interferon-alpha induction. *Cell. Mol. Immunol.* 7 263–270. 10.1038/cmi.2010.25 20473322 PMC4003230

[B21] Le Sage V. Banfield B. W. (2012). Dysregulation of autophagy in murine fibroblasts resistant to HSV-1 infection. *PLoS One* 7:e42636. 10.1371/journal.pone.0042636 22900036 PMC3416809

[B22] Lee H. K. Lund J. M. Ramanathan B. Mizushima N. Iwasaki A. (2007). Autophagy-dependent viral recognition by plasmacytoid dendritic cells. *Science* 315 1398–1401. 10.1126/science.1136880 17272685

[B23] Lee H. K. Mattei L. M. Steinberg B. E. Alberts P. Lee Y. H. Chervonsky A. (2010). In vivo requirement for Atg5 in antigen presentation by dendritic cells. *Immunity* 32 227–239. 10.1016/j.immuni.2009.12.006 20171125 PMC2996467

[B24] Levine B. (2005). Eating oneself and uninvited guests: autophagy-related pathways in cellular defense. *Cell* 120 159–162. 10.1016/j.cell.2005.01.005 15680321

[B25] Levine B. Mizushima N. Virgin H. W. (2011). Autophagy in immunity and inflammation. *Nature* 469 323–335. 10.1038/nature09782 21248839 PMC3131688

[B26] Liang Q. Seo G. J. Choi Y. J. Kwak M.-J. Ge J. Rodgers M. A. (2014). Crosstalk between the cGAS DNA sensor and Beclin-1 autophagy protein shapes innate antimicrobial immune responses. *Cell Host Microbe* 15 228–238. 10.1016/j.chom.2014.01.009 24528868 PMC3950946

[B27] Liu Q. Zhou Y. H. Yang Z. Q. (2016a). The cytokine storm of severe influenza and development of immunomodulatory therapy. *Cell. Mol. Immunol.* 13 3–10. 10.1038/cmi.2015.74 26189369 PMC4711683

[B28] Liu W. J. Ye L. Huang W. F. Guo L. J. Xu Z. G. Wu H. L. (2016b). p62 links the autophagy pathway and the ubiqutin-proteasome system upon ubiquitinated protein degradation. *Cell. Mol. Biol. Lett.* 21:29. 10.1186/s11658-016-0031-z 28536631 PMC5415757

[B29] Liu Z. Xiao Y. Torresilla C. Rassart,É and Barbeau B. (2017). Implication of different HIV-1 genes in the modulation of autophagy. *Viruses* 9:E389. 10.3390/v9120389 29258265 PMC5744163

[B30] Looker K. J. Magaret A. S. May M. T. Turner K. M. Vickerman P. Gottlieb S. L. (2015). Global and regional estimates of prevalent and incident herpes simplex virus type 1 infections in 2012. *PLoS One* 10:e0140765. 10.1371/journal.pone.0140765 26510007 PMC4624804

[B31] Lu Q. Yokoyama C. C. Williams J. W. Baldridge M. T. Jin X. DesRochers B. (2016). Homeostatic control of innate lung inflammation by Vici syndrome gene Epg5 and additional autophagy genes promotes influenza pathogenesis. *Cell Host Microbe* 19 102–113. 10.1016/j.chom.2015.12.011 26764600 PMC4714358

[B32] Lupfer C. Thomas P. G. Anand P. K. Vogel P. Milasta S. Martinez J. (2013). Receptor interacting protein kinase 2–mediated mitophagy regulates inflammasome activation during virus infection. *Nat. Immunol.* 14 480–488. 10.1038/ni.2563 23525089 PMC3631456

[B33] Lussignol M. Queval C. Bernet-Camard M.-F. Cotte-Laffitte J. Beau I. Codogno P. (2013). The herpes simplex virus 1 Us11 protein inhibits autophagy through its interaction with the protein kinase PKR. *J. Virol.* 87 859–871. 10.1128/JVI.01158-12 23115300 PMC3554085

[B34] Maartens G. Celum C. Lewin S. R. (2014). HIV infection: epidemiology, pathogenesis, treatment, and prevention. *Lancet* 384 258–271. 10.1016/S0140-6736(14)60164-1 24907868

[B35] McFarlane S. Aitken J. Sutherland J. S. Nicholl M. J. Preston V. G. Preston C. M. (2011). Early induction of autophagy in human fibroblasts after infection with human cytomegalovirus or herpes simplex virus 1. *J. Virol.* 85 4212–4221. 10.1128/JVI.02435-10 21325419 PMC3126239

[B36] Mizushima N. Yoshimori T. Ohsumi Y. (2011). The role of Atg proteins in autophagosome formation. *Annu. Rev. Cell Dev. Biol.* 27 107–132. 10.1146/annurev-cellbio-092910-154005 21801009

[B37] Moscona A. (2005). Neuraminidase inhibitors for influenza. *N. Engl. J. Med.* 353 1363–1373. 10.1056/NEJMra050740 16192481

[B38] Münz C. (2017). Autophagy proteins in viral exocytosis and anti-viral immune responses. *Viruses* 9:288. 10.3390/v9100288 28976939 PMC5691639

[B39] Nardacci R. Amendola A. Ciccosanti F. Corazzari M. Esposito V. Vlassi C. (2014). Autophagy plays an important role in the containment of HIV-1 in nonprogressor-infected patients. *Autophagy* 10 1167–1178. 10.4161/auto.28678 24813622 PMC4203545

[B40] Orvedahl A. Alexander D. Tallóczy Z. Sun Q. Wei Y. Zhang W. (2007). HSV-1 ICP34.5 confers neurovirulence by targeting the beclin 1 autophagy protein. *Cell Host Microbe* 1 23–35. 10.1016/j.chom.2006.12.001 18005679

[B41] Orvedahl A. MacPherson S. Sumpter R. Tallóczy Z. Zou Z. Levine B. (2010). Autophagy protects against sindbis virus infection of the central nervous system. *Cell Host Microbe* 7 115–127. 10.1016/j.chom.2010.01.007 20159618 PMC2860265

[B42] Orvedahl A. Sumpter R. Xiao G. Ng A. Zou Z. Tang Y. (2011). Image-based genome-wide siRNA screen identifies selective autophagy factors. *Nature* 480 113–117. 10.1038/nature10546 22020285 PMC3229641

[B43] Paludan C. Schmid D. Landthaler M. Vockerodt M. Kube D. Tuschl T. (2005). Endogenous MHC Class II processing of a viral nuclear antigen after autophagy. *Science* 307 593–596. 10.1126/science.1104904 15591165

[B44] Paul P. Münz C. (2016). Autophagy and mammalian viruses. *Adv. Virus Res.* 95 149–195. 10.1016/bs.aivir.2016.02.002 27112282

[B45] Paules C. Subbarao K. (2017). Influenza. *Lancet* 390 697–708. 10.1016/S0140-6736(17)30129-0 28302313

[B46] Peiris J. Hui K. P. Yen H.-L. (2010). Host response to influenza virus: protection versus immunopathology. *Curr. Opin. Immunol.* 22 475–481. 10.1016/J.COI.2010.06.003 20594815 PMC2927395

[B47] Perot B. P. Boussier J. Yatim N. Rossman J. S. Ingersoll M. A. Albert M. L. (2018). Autophagy diminishes the early interferon-β response to influenza A virus resulting in differential expression of interferon-stimulated genes. *Cell Death Dis.* 9:539. 10.1038/s41419-018-0546-5 29748576 PMC5945842

[B48] Pilli M. Arko-Mensah J. Ponpuak M. Roberts E. Master S. Mandell M. A. (2012). TBK-1 promotes autophagy-mediated antimicrobial defense by controlling autophagosome maturation. *Immunity* 37 223–234. 10.1016/j.immuni.2012.04.015 22921120 PMC3428731

[B49] Radtke K. English L. Rondeau C. Leib D. Lippé R. Desjardins M. (2013). Inhibition of the host translation shutoff response by herpes simplex virus 1 triggers nuclear envelope-derived autophagy. *J. Virol.* 87 3990–3997. 10.1128/JVI.02974-12 23365427 PMC3624196

[B50] Randow F. MacMicking J. D. James L. C. (2013). Cellular self-defense: how cell-autonomous immunity protects against pathogens. *Science* 340 701–706. 10.1126/science.1233028 23661752 PMC3863583

[B51] Rasmussen S. B. Horan K. A. Holm C. K. Stranks A. J. Mettenleiter T. C. Simon A. K. (2011). Activation of autophagy by α-herpesviruses in myeloid cells is mediated by cytoplasmic viral DNA through a mechanism dependent on stimulator of IFN genes. *J. Immunol.* 187 5268–5276. 10.4049/jimmunol.1100949 21998456 PMC3208073

[B52] Sagnier S. Daussy C. F. Borel S. Robert-Hebmann V. Faure M. Blanchet F. P. (2015). Autophagy restricts HIV-1 infection by selectively degrading Tat in CD4 + T lymphocytes. *J. Virol.* 89 615–625. 10.1128/JVI.02174-14 25339774 PMC4301118

[B53] Saitoh T. Fujita N. Hayashi T. Takahara K. Satoh T. Lee H. (2009). Atg9a controls dsDNA-driven dynamic translocation of STING and the innate immune response. *Proc. Natl. Acad. Sci. U.S.A.* 106 20842–20846. 10.1073/pnas.0911267106 19926846 PMC2791563

[B54] Schmid D. Pypaert M. Münz C. (2007). Antigen-loading compartments for major histocompatibility complex class II molecules continuously receive input from autophagosomes. *Immunity* 26 79–92. 10.1016/j.immuni.2006.10.018 17182262 PMC1805710

[B55] Sparrer K. M. J. Gableske S. Zurenski M. A. Parker Z. M. Full F. Baumgart G. J. (2017). TRIM23 mediates virus-induced autophagy via activation of TBK1. *Nat. Microbiol.* 2 1543–1557. 10.1038/s41564-017-0017-2 28871090 PMC5658249

[B56] Stolz A. Ernst A. Dikic I. (2014). Cargo recognition and trafficking in selective autophagy. *Nat. Cell Biol.* 16 495–501. 10.1038/ncb2979 24875736

[B57] Tal M. C. Sasai M. Lee H. K. Yordy B. Shadel G. S. Iwasaki A. (2009). Absence of autophagy results in reactive oxygen species-dependent amplification of RLR signaling. *Proc. Natl. Acad. Sci. U.S.A.* 106 2770–2775. 10.1073/pnas.0807694106 19196953 PMC2650341

[B58] Tallóczy Z. Virgin H. W. Levine B. (2006). PKR-dependent autophagic degradation of herpes simplex virus type 1. *Autophagy* 2 24–29. 16874088 10.4161/auto.2176

[B59] Taylor G. S. Long H. M. Brooks J. M. Rickinson A. B. Hislop A. D. (2015). The immunology of Epstein-Barr virus–induced disease. *Annu. Rev. Immunol.* 33 787–821. 10.1146/annurev-immunol-032414-112326 25706097

[B60] Weidberg H. Elazar Z. (2011). TBK1 mediates crosstalk between the innate immune response and autophagy. *Sci. Signal.* 4:pe39. 10.1126/scisignal.2002355 21868362

[B61] Whitley R. J. Roizman B. (2001). Herpes simplex virus infections. *Lancet* 357 1513–1518. 10.1016/S0140-6736(00)04638-9 11377626

[B62] Wild P. Farhan H. McEwan D. G. Wagner S. Rogov V. V. Brady N. R. (2011). Phosphorylation of the autophagy receptor optineurin restricts *Salmonella* growth. *Science* 333 228–233. 10.1126/science.1205405 21617041 PMC3714538

[B63] Yakoub A. M. Shukla D. (2015). Autophagy stimulation abrogates herpes simplex virus-1 infection. *Sci. Rep.* 5:9730. 10.1038/srep09730 25856282 PMC4929686

[B64] Yordy B. Iijima N. Huttner A. Leib D. Iwasaki A. (2012). A neuron-specific role for autophagy in antiviral defense against herpes simplex virus. *Cell Host Microbe* 12 334–345. 10.1016/j.chom.2012.07.013 22980330 PMC3454454

[B65] Zhou D. Kang K. H. Spector S. A. (2012). Production of interferon by human immunodeficiency virus type 1 in human plasmacytoid dendritic cells is dependent on induction of autophagy. *J. Infect. Dis.* 205 1258–1267. 10.1093/infdis/jis187 22396599 PMC3308911

